# Knowledge of and Adherence to Anaemia Prevention Strategies among Pregnant Women Attending Antenatal Care Facilities in Juaboso District in Western-North Region, Ghana

**DOI:** 10.1155/2020/2139892

**Published:** 2020-08-01

**Authors:** Prince Kubi Appiah, Daniel Nkuah, Duut Abdulai Bonchel

**Affiliations:** ^1^Department of Family and Community Health, School of Public Health, University of Health and Allied Sciences, Ho, Ghana; ^2^Centre for Migration Studies, University of Ghana, Legon, Accra, Ghana

## Abstract

**Background:**

Anaemia in pregnancy is a major problem in both developed and developing countries. The commonest source of anaemia is nutritional deficiency of iron with evidence suggesting that up to 90% of maternal anaemia may be due to inadequate consumption of dietary iron; however, there are other causes which include worm infestation, HIV infection, and genetic disorders. There are some implemented approaches in Ghana including education and awareness creation, nutritional supplements, and control and prevention of parasitic infections among others to prevent and control anaemia in pregnancy. This study assessed pregnant women adherence to Ghana's anaemia prevention strategies being implemented in the Juaboso District.

**Method:**

A descriptive cross-sectional data on knowledge of and adherence to anaemia prevention strategies among pregnant women was collected. Pearson's chi-square and logistic regression models were used to assessed associations between predictor and outcome variables. A *p* value <0.05 was considered as statistically significant. *Findings*. About 13.5% of the pregnant women had high knowledge on anaemia, while 58.4% and 28.1% had moderate and low knowledge, respectively. Less than half (39.1%) of the women adhered to anaemia prevention strategies. There were significant associations between knowledge of anaemia and where pregnant woman resides in the district (AOR: 2.04, 95% CI: 2.16-9.83, *p* = 0.003), woman's educational (AOR: 10.43, 95% CI: 6.14-51.63, *p* = 0.002), and occupational status (AOR: 15.14, 95% CI: 13.57-18.43, *p* < 0.001). Again, there were significant associations between adherence to anaemia prevention strategies and the woman's ethnicity (AOR: 0.61, 95% CI: 0.04-0.92, *p* = 0.001) and her knowledge of anaemia (AOR: 3.88, 95% CI: 1.32-7.93, *p* = 0.001).

**Conclusions:**

Pregnant women's knowledge of anaemia and adherence to anaemia prevention strategies are not encouraging. However, anaemia in pregnancy and its consequences could be devastating to all stakeholders if actions are not taken to reduce the phenomenon. Therefore, we recommend that more education and sensitisation programs including good nutritional practices in the diet of pregnant women be promoted to increase awareness and adherence to anaemia prevention strategies among pregnant women in the Juaboso District.

## 1. Introduction

Anaemia is one of the topmost causes of death globally and has been of a grave public health worry for both developing and developed countries affecting people of different age groups [1]. However, it is more prevalent in pregnant women, young children, and other women in reproductive age [2]. Globally, anaemia prevalence is about 29% in nonpregnant women, 38% in pregnant women, and 43% in children with the highest prevalence in South Asia and Central and West Africa [3]. The commonest cause of anaemia is iron deficiency with evidence suggesting that up to 90% of maternal anaemia are due to inadequate intake of dietary iron. However, worm infestations (hookworm and schistosomiasis), bleeding haemorrhoids, vitamin B6 and B12 deficiencies, human immunodeficiency virus (HIV) infection, and genetic disorders such as sickle cell anaemia are other factors that cause anaemia in pregnancy [4, 5]. A cry of a baby immediately after birth gives joy to the mother. This means that a healthy mother and child after delivery is the ultimate outcome that the pregnant mother, her family, and the entire community expect. However, anaemia is associated with increased maternal and newborn health problems as well as death [6]. Ghana through the Ministry of Health has been at the forefront with interventions and strategies to control anaemia in pregnancy. These strategies include education and awareness creation, nutrient (iron) supplementation, and control and prevention of parasitic infections in pregnancy. Additionally, the use of insecticide-treated nets (ITNs) and intermittent preventive treatment (IPT) against malaria, effective deworming, and provision of improved water, sanitation, and hygiene services are also being implemented to prevent anaemia among pregnant women. These strategies are meant to address common preventive causes of anaemia such as iron deficiency, worm infestation, and malaria control in the country. However, data available indicates that 44.6% of pregnant women in Ghana are anaemic [7]. In view of this, the study examined pregnant women adherence to Ghana's anaemia prevention strategies being implemented in the Juaboso District.

## 2. Methods

### 2.1. Study Site

The study was conducted in the Juaboso District, which is located in the Western-North Region, Ghana. The district has an estimated population of about 173,878 and annual population growth rate of 2%, with a landmass of 1,284 km with 99 communities [8]. The district has 25 health facilities including a hospital, 12 Community-based Health Planning and Services (CHPS) Compounds, 5 clinics, and 7 maternity homes providing health care services including information, education, and communication (IE&C) to the people. Economic activities in the district includes farming, trading, and rearing of livestock. The major crops cultivated in the area include cocoa, rice, cassava, yam, and maize. Tomatoes, garden eggs, and pepper are the abundant vegetables in the area. The district has two major markets for trading activities. The district also has 4 senior secondary schools, 33 junior secondary schools, and 75 primary schools providing formal education to people within and outside the district, with no tertiary institution [9].

### 2.2. Study Population

The study population comprised pregnant women in the district. However, only those who have stayed in the district for three or more months, have registered, and were attending antenatal clinics (ANC) were willing to be part of the study and agreed to sign the informed consent forms that were included in the study. Non-Ghanaians, even if they had stayed up to the required period of time, as well as health professionals, and those who were severely ill were excluded.

### 2.3. Study Design

Descriptive cross-sectional study and quantitative method of data collection was used to collect data from pregnant women on adherence to Ghana's anaemia prevention strategies being implemented in the Juaboso District.

### 2.4. Sample Size

Five hundred and ninety-eight (598) participants were involved; this was determined using the formula: **n** = **z**^2^ × **p** (**q**) ÷ **d**^2^ [10]. Where **n** is the sample size to be determined, **z** is the z-score (reliability coefficient) of 1.96 at 95% confidence level (C.L), **p** is the prevalence of anaemia among pregnant women in Ghana 44.6% [7], **q** is 1-*p*, and **d** is the degree of accuracy desired. With the extra implausibility about the true prevalence of anaemia among pregnant women due to a cluster sample survey design, design effect was considered in the sample size calculation. Therefore, the sample size became *n* × design effect (which was 1.5 in this case) (379.68 × 1.5 = 569.52). For a 5% nonresponse rate of 569.52, the sample size was upwardly adjusted and rounded to 598. This sample size ensured, with a probability of 95%, that the estimated prevalence was within ±5% of the true population coverage.

### 2.5. Sampling Method

Different (multistage) sampling techniques were used to select respondents. The district was stratified into the existing number (4) of subdistricts. A list of all ANC centres with registered pregnant women from each stratum was obtained from the district health directorate. A sample size was proportionately allocated to each stratum based on the list of pregnant women who have registered and were attending ANC. For each stratum, 2 ANC centres were randomly selected, and based on the sample size calculated for each stratum and the total population of pregnant women who have registered at each of the selected ANC centre, proportionate allocation was again used to allocate sample size to each selected ANC centre. With reference to the sample size for each ANC centre, special numbers were assigned to all registered women and randomly selected the required respondents.

### 2.6. Data Collection Tool and Procedure

Data was collected through administration of a semistructured questionnaire using a face-to-face interview technique. The questionnaire was pretested on 20 pregnant women with similar characteristics of the study participants from adjacent district for necessary modifications before being administered to the study participants. Data collection tool was a semistructured questionnaire comprised of demographic characteristics of the participants, women's knowledge of anaemia (causes, signs and symptoms, and available preventive strategies), and adherence to anaemia preventive strategies sections. Participants' ages were accessed using their birth certificates and ANC record cards. Participants contact information obtained from the facilities were used to trace to their homes and residence for data collection. Field assistants used these methods and tools to collect data from the pregnant women between May and June 2019. Data collected from each participant averaged 20 minutes.

Knowledge of anaemia was assessed based on 22 questions with 22 scores; pregnant women who scored 0-7 points were considered as having low knowledge of anaemia; those who scored 8-15 point were also considered as having fair knowledge of anaemia, while those who scored 16-22 points were classified as having high knowledge of anaemia. Seven questions with 7 points were used to assess adherence to anaemia prevention strategies; pregnant women who scored 6 or less points were considered as partially adhering, while those who scored all the 7 points were considered as completely adhering to anaemia preventive strategies.

### 2.7. Ethical Issues

The study conformed to the required ethical regulations regarding the use of humans and was approved by the Ethical Review Committee of the Ghana Health Services, Research and Development Division, Accra with protocol number GHS-ERC 150/05/17. Participation in the study was voluntary; consent and assent were sought from the participants and guardians after the study processes had been explained to them.

### 2.8. Data Analysis

Double data entry was performed and checked for completeness and consistency using Epi data version 3.1. and Stata version 13 for data analysis, with illustrations in tables and graphs. In addition to descriptive statistics, associations between dependent and independent variables were analysed using Pearson's chi^2^ and multiple (univariate and multivariate) logistic regression models. A *p* value <0.05 was considered as statistically significant.

## 3. Results

### 3.1. Demographic Characteristics of Participants

A total of 598 pregnant women were involved in the study with mean age of 24.4 years (±2.6 sd), and most (44.3%) of them were 20-29 years old. A comparative majority (27.3%) of them were from Juaboso subdistrict. About 18.2% of the women never went to school. The majority (78.6%) of the pregnant women were legally married, 68.7% of them were Christians, while 56.2% were Akans. Again, the majority (67.9%) of the pregnant women were involved in nonformal jobs, while 19.9% of them were unemployed. Most (44.7%) of the women were in 2^nd^ trimester of gestational period, while 65.4% of them have had 1 pregnancy (parity 1) before the current pregnancy (Table 1).

### 3.2. Knowledge of Anaemia among Pregnant Women

**Figure 1:**
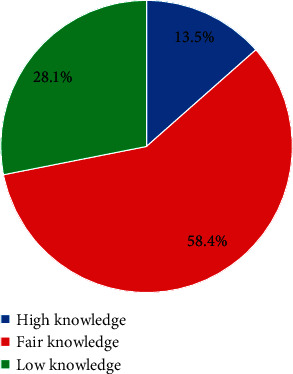
Knowledge of anaemia among pregnant women.

About 13.5% of the pregnant women had high knowledge of anaemia, while 58.4% and 28.1% of them had fair knowledge and low knowledge, respectively, (Figure 1).

### 3.3. Adherence to Anaemia Prevention Strategies among Pregnant Women

**Figure 2:**
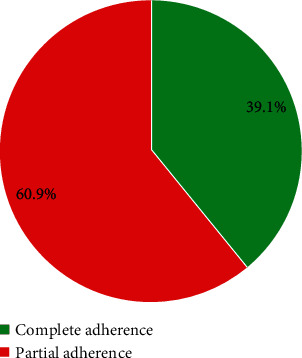
Adherence to anaemia prevention strategies among pregnant women.

The majority of the pregnant women were partially adhering to anaemia prevention strategies, 39.1% of them completely adhering to the preventive strategies (Figure 2).

### 3.4. Associations between Knowledge on Anaemia and General Characteristics

The study showed significant associations between knowledge of anaemia and the subdistrict where the pregnant woman resides (*p* = 0.003), the woman's educational status (*p* = 0.002), and occupational status (*p* ≤ 0.001).

Additionally, when the variable that showed associations with knowledge of anaemia from univariate analysis were tested for confounding effects using multivariate logistic regression analysis, it was confirmed that pregnant women who were residing in the Bonsu (AOR: 0.21, 95% CI: 2.31-8.81, *p* = 0.004), Jato (AOR: 3.06, 95% CI: 1.96-7.18, *p* = 0.002) and Asempaneye subdistricts (AOR: 2.04, 95% CI: 2.16-9.83, *p* = 0.003) were less likely to have high knowledge of anaemia than those who were residing in the Juaboso subdistrict. Also, pregnant women who attained basic (AOR: 1.78, 95% CI: 1.89-5.37, *p* = 0.001), secondary (AOR: 4.22, 95% CI: 2.23-9.16, *p* = 0.002), and tertiary education (AOR: 10.43, 95% CI: 6.14-15.63, *p* = 0.002) were more likely to have high knowledge of anaemia than women who never went to school. Again, women who were engaged in nonformal (AOR: 2.18, 95% CI: 1.07-6.69, *p* = 0.001) and formal jobs (AOR: 15.14, 95% CI: 13.57-18.43, *p* < 0.001) were more likely to have high knowledge of anaemia than those who were unemployed (Table 2).

### 3.5. Associations between Adherence to Anaemia Prevention Strategies and General Characteristics and Knowledge on Anaemia

The study revealed significant associations between adherence to anaemia prevention strategies and ethnicity of the pregnant woman (*p* = 0.001) and woman's knowledge of anaemia (*p* = 0.001).

Additionally, when the variable that showed associations with adherence to anaemia prevention strategies from univariate analysis were tested for confounding effects using multivariate logistic regression analysis, it was confirmed that pregnant women who were Ewes (AOR: 0.68, 95% CI: 0.02-0.87, *p* = 0.001), Ga-Adangbe (AOR: 0.53, 95% CI: 0.09-0.90, *p* = 0.002), and Kussase (AOR: 0.61, 95% CI: 0.04-0.92, *p* = 0.001) were less likely to adhere to anaemia prevention strategies than women who were Akans. Also, pregnant women who had high knowledge of anaemia (AOR: 3.88, 95% CI: 1.32-7.93, *p* = 0.001) were more likely to adhere to anaemia prevention strategies than women who had low or fair knowledge of anaemia prevention strategies (Table 3).

## 4. Discussion

This study showed that 18.2% of the pregnant women had never been to school, while 19.9% of them were unemployed. However, a study in Libya revealed that only 1.7% of pregnant women in the country were not educated; though, there is a civil war in the country that might have affected the educational system of the country [11], while about 19% and 9.9% of pregnant women in Nigeria and Uyo State, respectively, had never been to school [12, 13]. The findings of this study are similar to what was reported (19.1%) in the Ghana demographic and health survey [7]. The unemployment status reported in this study is lower than what was reported in the Southern Ghana, Kenyan, and South African studies [14–16].

This study showed that 86.5% of the pregnant women had insufficient (low/fair) knowledge of anaemia. This finding is in contrast with a study conducted among pregnant women in Nepal, which revealed that 56% of the women had insufficient knowledge of anaemia [17]. Also, a study to assess the knowledge and risk factors of anaemia among pregnant women in Libya revealed that all of the women had moderate knowledge on anaemia [11]. Again, a study conducted in the Brosankro in Ghana reported that less than 30% of pregnant women knew signs and symptoms of anaemia [18]. The present study indicates that a significant number of the pregnant women knew that treatment of worm infestation can help prevent anaemia. This is in contrast to the lower finding reported in the Nepal and Nigeria studies [19, 20]. The reason for the differences in the treatment of worm infestation as one of the anaemia prevention strategies could be the sample size of the studies. The use of insecticide-treated nets (ITNs) has been recommended as an integral part of maternal and child health policies in Sub-Saharan Africa where malaria infection is endemic and a major cause of severe anaemia in pregnancy [21, 22]. Therefore, it is not surprising that most of the pregnant women in the current study knew that sleeping under ITNs prevents malaria and is an anaemia prevent strategy, which agrees with the Nigerian study [20]. Though, a good proportion of the pregnant women were aware that the use of ITNs is a strategy to prevent anaemia in pregnancy; this awareness should be sustained, and efforts towards achieving 100% awareness should be enhanced.

Adherence to anaemia prevention strategies plays a major role in the prevention and treatment of anaemia particularly among pregnant women whose iron requirement increases at the second trimester and progresses until the third trimester [23, 24]. Generally, the current study revealed that only 39.1% of the pregnant women were fully adhering to anaemia prevention strategies. This finding is similar to a study conducted in Kathmandu, Nepal, which revealed that the majority of pregnant women did not adhere to practices required to prevent anaemia in pregnancy [19]. However, the finding of the present study is in contrast with a study conducted among pregnant women in Mecha district, Western Ethiopia [25]. This discrepancy could be that the Ethiopia study was based on pregnant women who took iron folate tablets for 90 or more days during the entire pregnancy, while this study was based on pregnant women who took iron folate supplement within the entire duration of the pregnancy. Moreover, the probable reason may be the difference in geographical locations and accessibility of health institutions in these countries.

There was a strong statistically significant association between adherence to anaemia prevention strategies and woman's knowledge of anaemia and showed that pregnant women who had high knowledge were completely adhering to anaemia prevention strategies as compared to those who had poor knowledge. This association is an indication that the level of knowledge significantly contributed to the level of adherence. What it means is that nonadherence occurs as a result of ignorance and inadequate knowledge a pregnant woman has about anaemia. Consequently, sustained education of pregnant women on anaemia and its preventive strategies are central to maximize adherence to anaemia prevention strategies. Also, these findings are in harmony with a study conducted in Mecha district, Western Amhara in Ethiopia [25]. Again, pregnant woman's ethnicity was significantly associated with adherence to anaemia prevention strategies. This finding agrees with Barroso et al. and the Belgium studies [26, 27].

This study also revealed significant associations between where a pregnant woman resides in the district, woman's educational, and occupational status. The finding indicated that pregnant women who went to school were more likely to have high knowledge of anaemia than women who never went to school. This revelation agrees with the studies in India and Pakistan [28–30]. Again, the current study showed that pregnant women who had jobs were more likely to be associated with high knowledge of anaemia as compared to women who were not employed, with a study showing that people who have low economic are more likely to suffer from anaemia [31]. This could be due to the fact that education level attained is required in job seeking, which in turn increases earning power of those employed and also increases their number of ANC visits at the health facilities where education on anaemia prevention is always given.

## 5. Conclusion

Knowledge of anaemia and adherence to anaemia prevention strategies among pregnant women in the district were generally not encouraging. This trend if continued could hinder efforts to reduce anaemia in pregnancy in the country, as well as preventing the country from achieving the targets of Sustainable Development Goals 3. Hence, more efforts are needed to promote awareness on and adherence to anaemia prevention in the district as anaemia in pregnancy could be detrimental to both mother and the foetus, as well as the community and the country as a whole.

### 5.1. Recommendations

- The presence of low knowledge and adherence to anaemia prevention suggests the need for an intensification of education on anaemia and its prevention strategies by health professionals and collaborators at all levels of health delivery services to all women in reproductive age. This should include more education and sensitisation on good nutritional practices in the diet of pregnant women
- Early childhood education on anaemia and other health conditions among women and children should be encouraged and instituted in educational curriculum to offer them with knowledge on important health issues before they reach adulthood, this should be done in collaboration with Ghana Education Service, Ghana Health Service, and other relevant agencies
- Male involvement and active participation in women and child health issues should be encouraged and promoted since women need support of all people to be able to adhere to all health promotion strategies. This can be achieved through durbars to highlight the roles and duties of men in women's health and also to establish awards for men who accompany their spouses to antenatal clinics and other health facilities for healthcare.

## Figures and Tables

**Table 1:** Demographic characteristics of participants.

Variable		Frequency	Percentage
Subdistrict	Juaboso	163	27.3
	Bonsu	152	25.4
	Jato	139	23.2
	Asempaneye	144	24.1
Age (years)	10–19	72	12.0
	20–29	265	44.3
	30–39	194	32.5
	≥40	67	11.2
Educational status	None	109	18.2
	Basic (primary)	261	43.7
	Secondary	155	25.9
	Tertiary	73	12.2
Marital status	Legally married	470	78.6
	Cohabitation	128	21.4
Religion	Christianity	411	68.7
	Islam	120	20.1
	Traditionalist	67	11.2
Ethnicity	Akan	336	56.2
	Ewe	79	13.2
	Ga-Adangbe	63	10.5
	Kussase	120	20.1
Occupation	Unemployed	119	19.9
	Nonformal jobs	406	67.9
	Formal jobs	73	12.2
Gestational age of the pregnancy	1^st^ trimester	146	24.4
	2^nd^ trimester	267	44.7
	3^rd^ trimester	185	30.9
Parity	0	51	8.5
	1	391	65.4
	2	101	16.9
	≥3	55	9.2

**Table 2:** Associations between knowledge of anaemia and demographic characteristics.

Variables	Knowledge of anaemia		OR (95% CI) *p* value		AOR (95% CI) *p* value
	Low/fair = 517 (86.5%)	High = 81 (13.5%)			
Subdistrict					
Juaboso	136 (83.4)	27 (16.6)	1	**0.003**	1
Bonsu	134 (88.2)	18 (11.8)	0.49 (2.01-8.95)		0.41 (2.31-8.81) **0.004**
Jato	116 (83.5)	23 (16.5)	0.71 (1.89-7.43)		0.76 (1.96-7.18) **0.002**
Asempaneye	131 (91.0)	13 (9.0)	0.23 (2.15-9.30)		0.24 (2.16-9.83) **0.003**
Age-group (years)					
10–19	53 (73.6)	19 (26.4)	1	0.258	
20–29	244 (92.1)	21 (7.9)	0.54 (0.04-7.10)		
30–39	172 (88.7)	22 (11.3)	0.67 (0.11-6.88)		
≥40	48 (71.6)	19 (28.4)	1.72 (0.02-3.48)		
Educational status					
None	105 (96.3)	4 (3.7)	1	**0.002**	1
Basic	240 (92.0)	21 (8.0)	2.34 (1.25-5.62)		1.78 (1.89-5.37) **0.001**
Secondary	126 (81.3)	29 (18.7)	4.23 (2.33-9.89)		4.22 (2.23-9.16) **0.002**
Tertiary	46 (63.0)	27 (37.0)	12.31 (10.24-17.98)		10.43 (6.14-15.63) **0.002**
Marital status					
Legally married	424 (90.2)	46 (9.8)	1	0.364	
Cohabitation	93 (72.7)	35 (27.3)	3.59 (1.03-7.23)		
Religion					
Christianity	375 (91.2)	36 (8.8)	1	0.347	
Muslim	94 (78.3)	26 (21.7)	5.23 (3.11-8.81)		
Traditionalist	48 (71.6)	19 (28.4)	7.72 (4.30-11.33)		
Ethnicity					
Akan	311 (92.6)	25 (7.4)	1	0.063	
Ewe	62 (78.5)	17 (21.5)	3.84 (0.51-6.61)		
Ga-Adangbe	45 (71.4)	18 (28.6)	4.57 (0.33-8.15)		
Kussase	99 (82.5)	21 (17.5)	2.46 (1.60-4.79)		
Occupation					
Unemployed	108 (90.8)	11 (9.2)	1	**<0.001**	1
Nonformal jobs	359 (88.4)	47 (11.6)	2.48 (2.04-6.37)		2.18 (1.07-6.69) **0.001**
Formal jobs	50 (68.5)	23 (31.5)	18.09 (5.42-25.28)		15.14 (13.57-18.43) **<0.001**
Gestational age of the pregnancy					
1^st^ trimester	133 (91.1)	13 (8.9)	1	0.736	
2^nd^ trimester	230 (86.1)	37 (13.9)	2.25 (0.84-6.17)		
3^rd^ trimester	154 (83.2)	31 (16.8)	2.62 (0.34-7.34)		
Parity					
0	47 (92.2)	4 (7.8)	1	0.682	
1	360 (92.1)	31 (7.9)	1.67 (0.34-8.76)		
2	74 (73.3)	27 (26.7)	3.74 (0.23-9.82)		
≥3	36 (65.5)	19 (34.5)	6.93 (0.19-9.14)		

**Table 3:** Associations between adherence to anaemia prevention strategies and general characteristics and knowledge on anaemia.

Variables	Adherence to anaemia prevention strategies		OR (95% CI) *p* value		AOR (95% CI) *p* value
	Partial adherence 364 (60.9)	Complete adherence 234 (39.1)			
Subdistrict					
Juaboso	98 (60.1)	65 (39.9)	1	0.123	
Bonsu	92 (60.5)	60 (39.5)	0.81 (0.03-8.76)		
Jato	83 (59.7)	56 (40.3)	1.78 (0.04-5.84)		
Asempaneye	91 (63.2)	53 (36.8)	0.65 (0.05-7.51)		
Age-group (years)					
10–19	61 (84.7)	11 (15.3)	1	0.237	
20–29	160 (60.4)	105 (39.6)	4.73 (0.26-8.47)		
30–39	101 (52.1)	93 (47.9)	6.65 (0.22-10.58)		
≥40	42 (62.7)	25 (37.3)	4.54 (0.01-9.29)		
Educational status					
None	98 (89.9)	11 (10.1)	1	0.371	
Basic	201 (77.0)	60 (23.0)	3.29 (0.23-7.67)		
Secondary	58 (37.4)	97 (62.6)	7.57 (0.11-11.32)		
Tertiary	7 (9.6)	66 (90.4)	8.24 (0.64-14.56)		
Marital status					
Legally married	262 (55.7)	208 (44.3)	1	0.421	
Cohabitation	102 (79.7)	26 (20.3)	0.48 (0.07-11.43)		
Religion					
Christianity	248 (60.3)	163 (39.7)	1	0.394	
Muslim	52 (43.3)	68 (56.7)	2.12 (0.03-4.45)		
Traditionalist	64 (95.5)	3 (4.5)	0.32 (0.02-11.41)		
Ethnicity					
Akan	181 (53.9)	155 (46.1)	1	**0.001**	1
Ewe	51 (64.6)	28 (35.4)	0.69 (0.01-0.89)		0.68 (0.02-0.87) **0.001**
Ga-Adangbe	53 (84.1)	10 (15.9)	0.51 (0.07-0.91)		0.53 (0.09-0.90) **0.002**
Kussase	79 (65.8)	41 (34.2)	0.67 (0.06-0.92)		0.61 (0.04-0.92) **0.001**
Occupation					
Unemployed	76 (63.9)	43 (36.1)	1	0.321	
Nonformal jobs	274 (67.5)	132 (32.5)	0.07 (0.13-2.43)		
Formal jobs	14 (19.2)	59 (80.8)	3.21 (0.28-7.25)		
Gestational age of the pregnancy					
1^st^ trimester	89 (61.0)	57 (39.0)	1	0.408	
2^nd^ trimester	169 (63.3)	98 (36.7)	0.72 (0.19-8.03)		
3^rd^ trimester	106 (57.3)	79 (42.7)	1.94 (0.21-3.67)		
Parity					
0	39 (76.5)	12 (23.5)	1	0.421	
1	280 (71.6)	111 (28.4)	1.67 (0.17-3.92)		
2	41 (40.6)	60 (59.4)	3.34 (0.62-4.27)		
≥3	4 (7.3)	51 (92.7)	7.56 (0.41-9.18)		
Knowledge of anaemia					
Low/fair	355 (68.7)	162 (31.3)	1	**0.001**	1
High	9 (11.1)	72 (88.9)	4.12 (2.76-8.66)		3.88 (1.32-7.93) **0.001**

## Data Availability

Answer: Yes. Comment: The data used to support the findings of this study can be made available from the corresponding author upon request.
